# Sezary Syndrome: A Case Report and Review of Current Therapeutics

**DOI:** 10.7759/cureus.58570

**Published:** 2024-04-18

**Authors:** Jayasree Krishnan, Kannan Thanikachalam

**Affiliations:** 1 Internal Medicine, Mobile Infirmary Medical Center, Mobile, USA; 2 Hematology/Oncology, Infirmary Health System, Mobile, USA; 3 Hematology/Oncology, Henry Ford Health System, Detroit, USA

**Keywords:** generalized skin rash, cutaneous t-cell lymphoma, leukemic, mycosis fungoides, sezary syndrome

## Abstract

Sezary syndrome (SS) is a rare but aggressive type of cutaneous T-cell lymphoma (CTCL). Patients with SS have characteristic skin lesions (erythroderma) and a leukemic phase. The rash associated with CTCLs can often mimic common benign skin conditions such as psoriasis, atopic dermatitis, etc. and therefore can go undiagnosed until later stages. We present a case of a patient with SS who managed eczema for over one year with topical steroids before receiving a skin biopsy. Workup confirmed leukemic involvement, and the patient was started on systemic therapy with bexarotene. The patient continues to have a good response to systemic therapy. When treating patients with persistent rash of uncertain etiology and/or unresponsive to treatment, primary care physicians and internists need to consider SS/Mycosis fungoides as a possible differential and should have a low threshold to initiate early referral to dermatology for definitive diagnosis.

## Introduction

Cutaneous T-cell lymphomas (CTCLs) are a rare group of lymphomas that primarily involve the skin. Mycosis fungoides (MF) is the most common type of CTCL accounting for approximately 57% of CTCLs [1]. MF presents with localized or widespread skin lesions (patch, plaque, or tumor) and in some cases, lymph nodes, blood, and viscera may also be involved [2]. Sezary syndrome (SS) is a rare type of CTCL characterized by erythroderma, generalized lymphadenopathy, and clonally related malignant T-cells in the skin and peripheral blood [2]. The incidence of SS/MF increases with age with the majority of cases over 40 years of age [1,2]. In the United States, the incidence is higher in men, non-Hispanic black patients, individuals in high socioeconomic status quintiles, and people living in metropolitan counties [1].

MF/SS can be challenging to diagnose since the skin lesions mimic inflammatory skin diseases like eczema and psoriasis, especially at an early stage. Increased awareness among physicians and education on heterogeneous presentation of this disease can lead to expedited specialist referrals and prompt diagnosis. Patients with early-stage MF/SS have favorable prognosis so early diagnosis and treatment are crucial to decrease the risk of progression and improve outcomes [3].

## Case presentation

**Figure 1:**
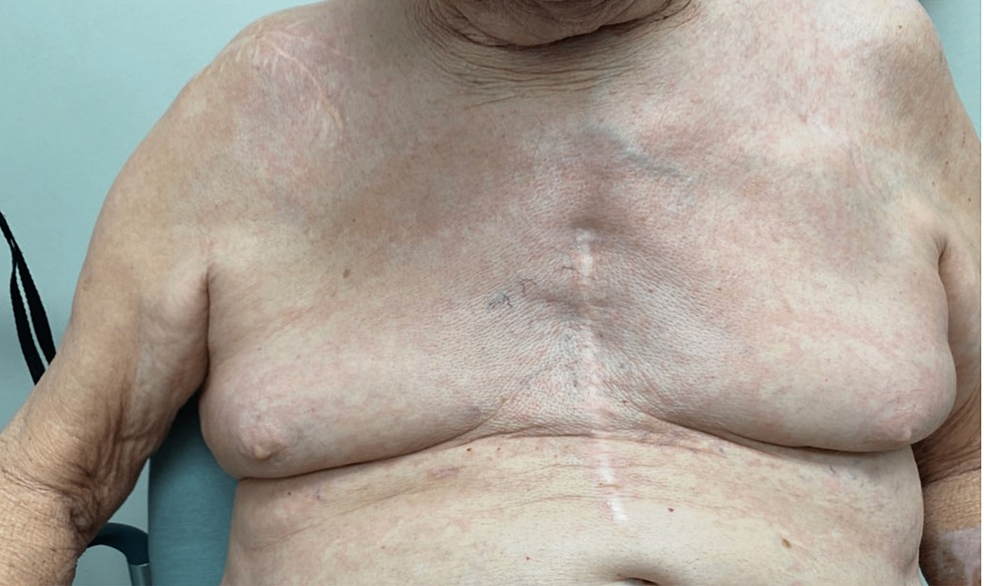
Erythematous skin rash in the chest wall before treatment

Our patient is a 79-year-old Caucasian gentleman with a past medical history of hypertension, hyperlipidemia, history of cardiac arrest from ventricular fibrillation s/p AICD requiring prolonged hospitalization who presented with a pruritic erythematous rash (Figure 1) all over his body. He was never diagnosed with any dermatological condition in the past. He denied any personal history or family history of malignancy. He is a former light smoker with five packs a year smoking history, with no alcohol use or recreational drug use. He had no drug or environmental allergies. On examination, the patient's vital signs were normal, with a temperature of 98.8, a heart rate of 90 beats/min, a respiratory rate of 18 breaths/min, and a blood pressure of 134/82. Cardiovascular, pulmonary, abdominal, and neurological exams were normal. Examination of the lymphatic system did not reveal any lymphadenopathy. A dermatological examination revealed an erythematous macular rash all over his body, predominantly in his chest, trunk, and lower extremities. Though he did not have any known history of eczema, given his pruritic nature, his erythematous rash was managed as possible eczema for nearly 1.5 years with topical steroids of varying strength by his primary care physician without any significant improvement in symptoms. The patient was subsequently referred to dermatology who performed a skin biopsy of epigastric skin lesion that came back as atypical T-cell infiltrate, consistent with MF. By Immunohistochemistry (IHC), the cells were uniformly positive for CD3 with a CD4: CD8 ratio of greater than 10-1 and diminished CD7 expression. CD20 stains showed rare, scattered cells. He was then referred to hematology for further evaluation. Peripheral blood flowcytometry showed an abnormal T cell population involving 26% of total leukocytes (CD45+, CD1a-, CD2+, sCD3+, CD4+, CD5+, CD7 -/+, CD8-, CD10-, CD11b-, CD11c-, CD13-, CD14-, CD16-, CD19-, CD20-, CD25 -/+, CD26-, CD30-, CD34-, CD38-/+, CD52+, CD56-, CD57-, CD103-, CD117-, TCRGD-, TRBC1+). The absolute Sezary cell count was 2.8K/mm^3^, consistent with low-intermediate burden disease. 

**Figure 2:**
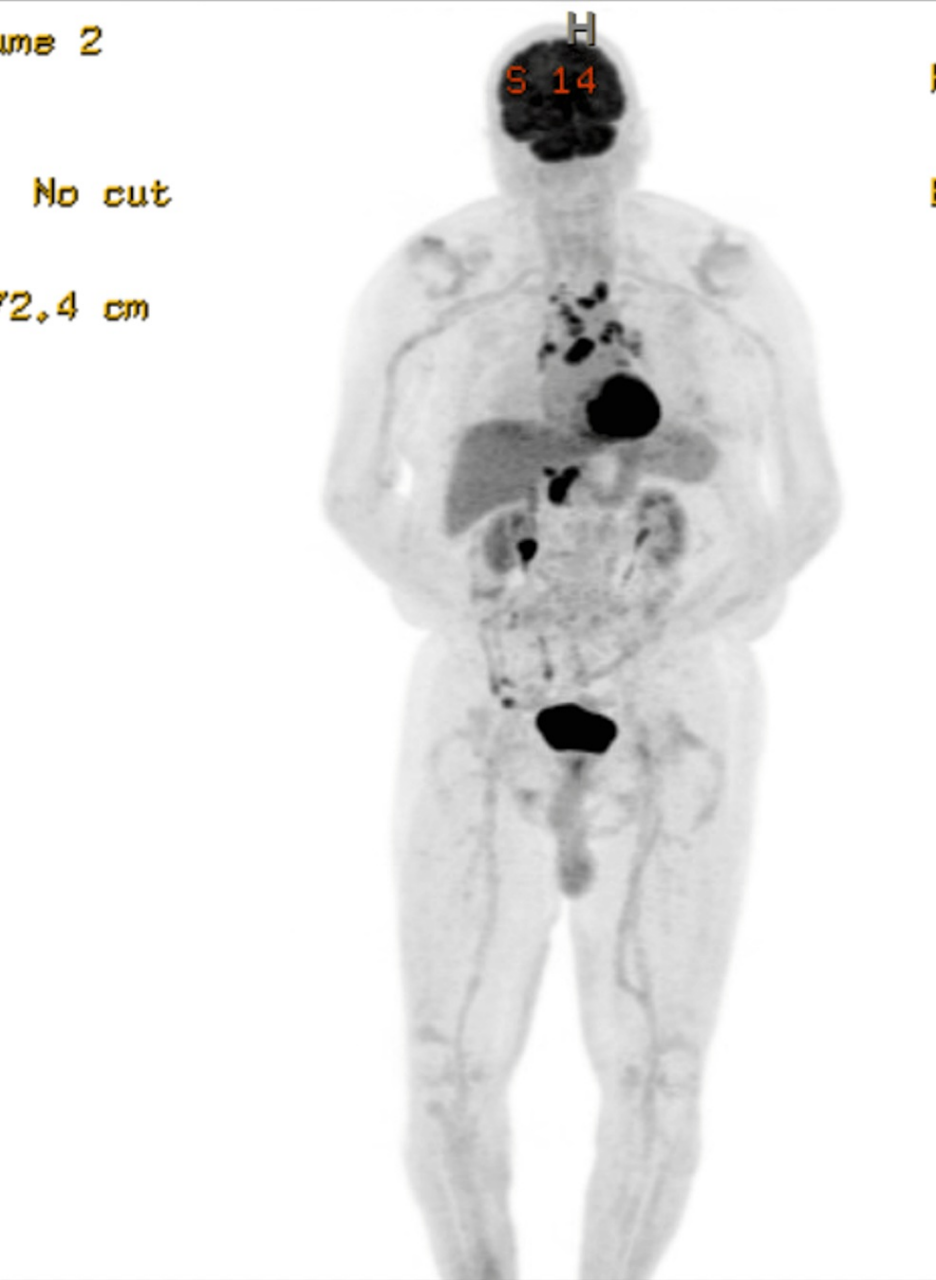
PET scan showing FDG avid lymphadenopathy in the mediastinum, hilar, and upper abdomen PET: Positron emission tomography; FDG: 18F-fluorodeoxyglucose

**Figure 3:**
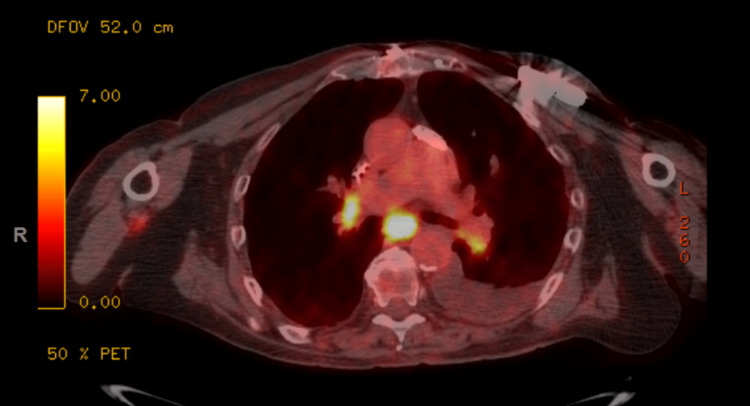
FDG-avid lymphadenopathy in the mediastinum FDG: 18F-fluorodeoxyglucose

**Figure 4:**
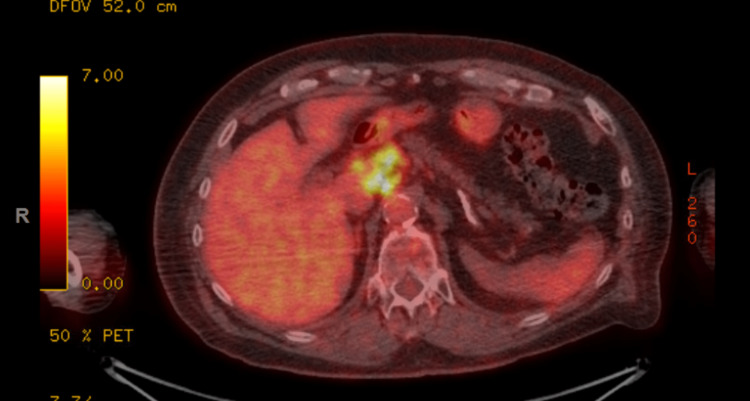
FDG-avid lymphadenopathy in the upper abdomen FDG: 18F-fluorodeoxyglucose

He underwent evaluation with bone marrow biopsy, positron emission tomography (PET) scan, and T-cell receptor (TCR) gene rearrangement clonality. Bone marrow biopsy showed normocellular bone marrow for age with trilineage hematopoiesis but with low-level involvement by SS. TCR gene rearrangement showed gamma chain clonal rearrangement. His PET scan showed 18F-fluorodeoxyglucose (FDG) avid lymphadenopathy in the mediastinum, hilar, and upper abdomen (Figures 2-4). Biopsy of FDG avid lymph nodes was not obtained given his advanced age, and comorbidities, and medical decision was made to monitor his response to therapy on subsequent imaging. Human T-cell lymphotropic virus (HTLV) serology was negative.

**Figure 5:**
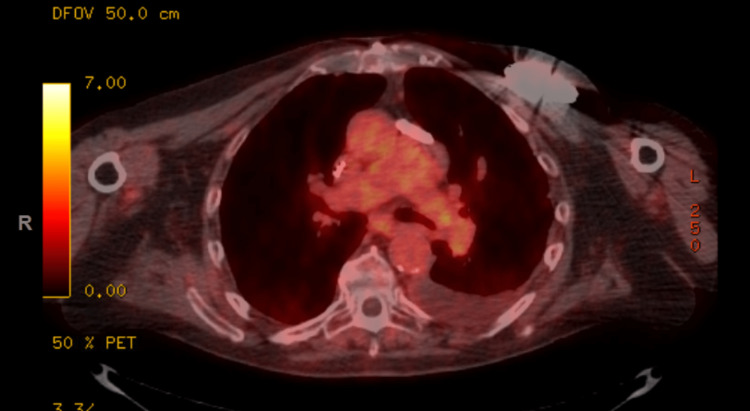
Interval improvement in FDG-avid mediastinal lymphadenopathy after two months of bexarotene treatment FDG: 18F-fluorodeoxyglucose

**Figure 6:**
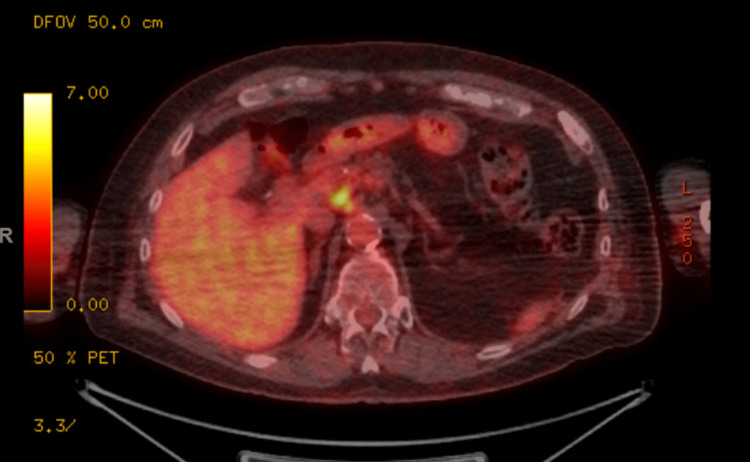
Interval improvement in FDG-avid abdominal lymphadenopathy after two months of bexarotene treatment FDG: 18F-fluorodeoxyglucose

**Figure 7:**
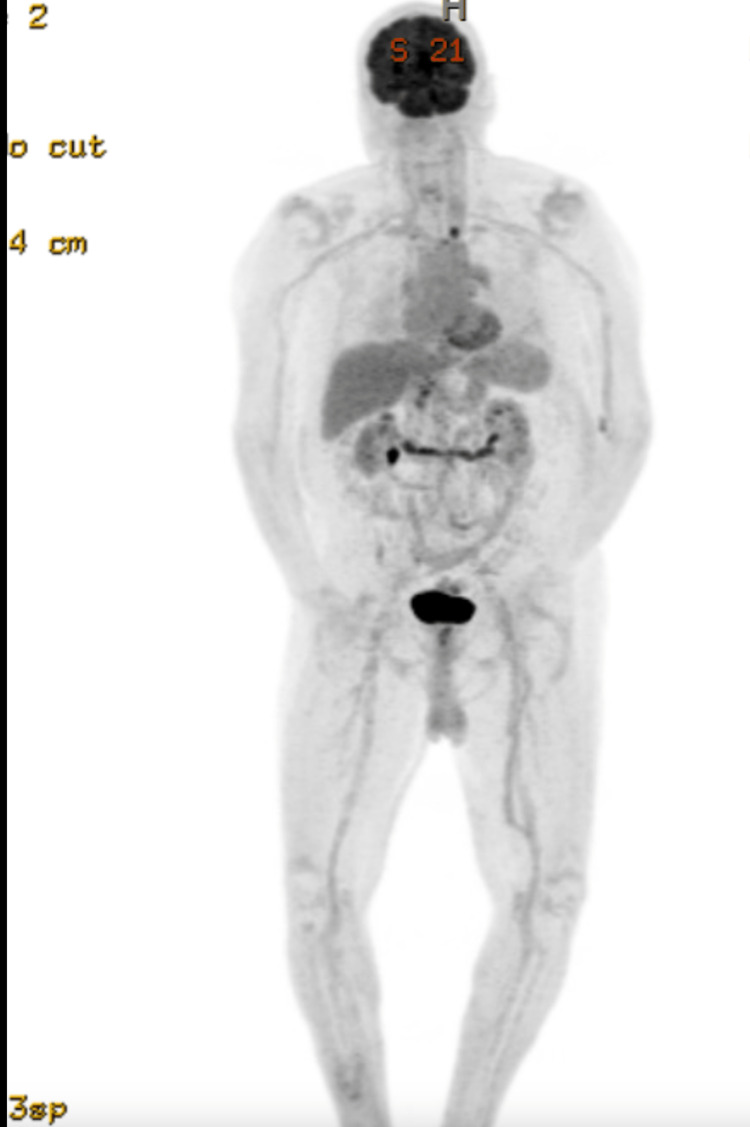
Radiological response after two months of treatment with bexarotene

**Figure 8:**
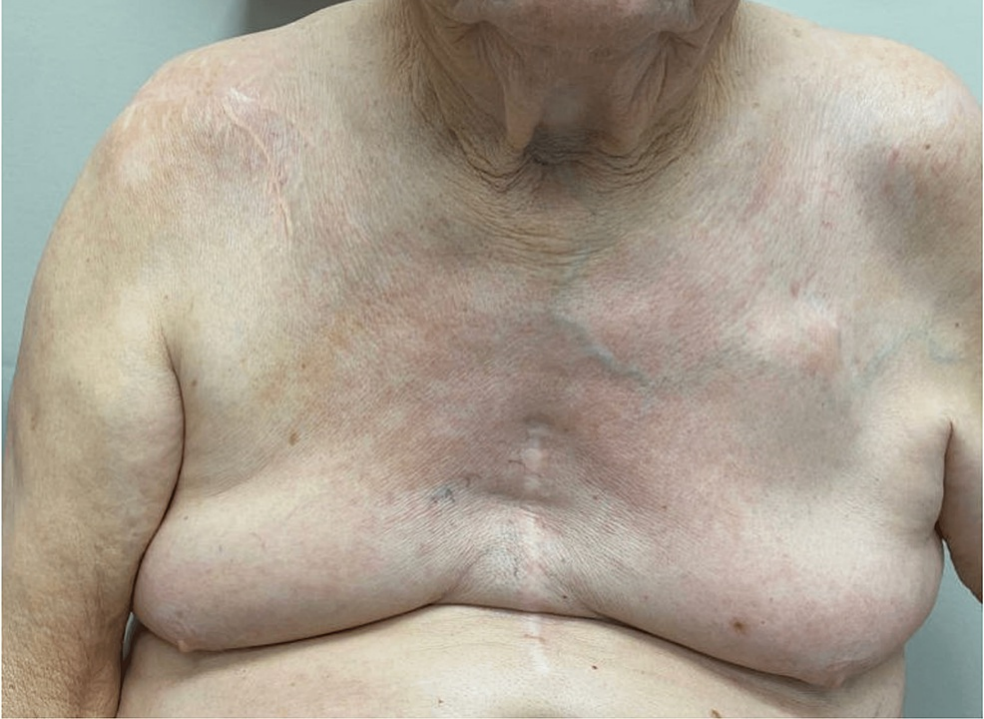
Improvement in erythematous skin rash one month after treatment

The patient was diagnosed with SS, stage IVA1 T4N3M0, B2 disease. He was started on systemic bexarotene, a retinoid, given low systemic toxicity and also received topical triamcinolone 0.05% cream for skin-directed therapy. The patient developed hypertriglyceridemia and hypothyroidism as a side effect of bexarotene which responded to an increase in the dose of atorvastatin and initiating low-dose levothyroxine respectively. He also experienced mild diarrhea which was well controlled with Imodium as needed. PET scan was repeated in two months after initiating treatment, which showed improvement in size and metabolic uptake (Figures 5-7). The largest intra-abdominal decreased in size from 2.3 cm to 1.7 cm with a decrease in the standardized uptake value (SUV) from 14.7 to 4.7. The largest mediastinal lymph node decreased in size from 3 cm to 1.2 cm with a decrease in SUV from 16 to 2. His skin rash improved with treatment as well (Figure 8). He remains stable without disease progression, after five months of bexarotene therapy.

## Discussion

SS and MF are the most common types of cutaneous T-cell lymphomas. The overall incidence of CTCL was 8.55 per million according to the data from the Surveillance, Epidemiology, and End Results (SEER) program from 2000 to 2018 which has increased from 6.4 per million persons during the period 1973-2002 [1,4]. The annual percentage change of CTCL was highest in Sezary syndrome with an increase of 3.83% during this period [1]. An increase in incidence is postulated due to better diagnostic tools and increased physician awareness however diagnostic delay persists due to the indolent nature of the disease, heterogeneous, nonspecific skin manifestations mimicking benign inflammatory dermatosis such as psoriasis, eczema, lichenoid dermatoses, etc.., leading to misdiagnosis. One study reported a mean diagnostic delay of 4.2 years overall with a delay of five years in patients without erythroderma at the initial visit [5]. 

**Figure 9:**
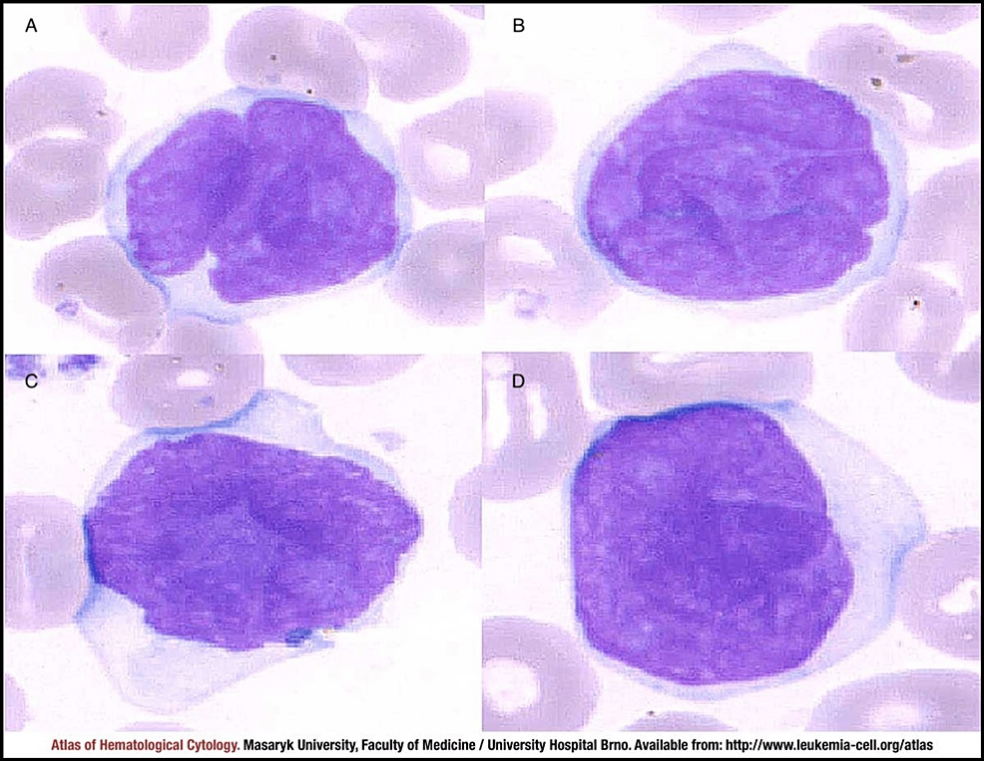
Peripheral blood smear of Sezary syndrome A-D: Typical morphology of Sezary cells with cerebriform nuclei [8]

The exact etiology of SS is unclear; however human T-lymphotropic viruses type 1 and 2 (HTLV-I/II) have been identified as a risk factor. Certain medications that may induce an antigen-driven T-cell lymphoproliferation or dyscrasia could serve as a trigger for MF [6]. SS may arise de novo or may precede MF. The diagnosis of SS is usually based on clinical, histopathological, and molecular findings. Since the skin and/or lymph node biopsy findings can be nonspecific and often not confirmatory but only suspicious for SS, the International Society for Cutaneous Lymphomas (ISCL) has proposed strict hematologic criteria to avoid misdiagnosis [7]. Sezary cells are characterized by variable amounts of non-granular cytoplasm along with the characteristic convoluted to cerebriform nuclei (Figure 9). The criteria for SS diagnosis require generalized erythroderma and the following hematologic criteria: 1. An absolute Sézary cell count of 1000 cells/mm^3^ or more 2. A CD4/CD8 ratio of 10 or more due to an increase in CD3+ or CD4+ cells by flow cytometry 3. Aberrant expression of pan-T cell markers (CD2, CD3, CD4, CD5) by flow cytometry. Deficient CD7 expression on T cells (or expanded CD4+CD7- cells ≥ 40%) represents a tentative criterion of SS at this time. 4. Increased lymphocyte counts with evidence of a T-cell clone in the blood by Southern blot or PCR technique 5. A chromosomally abnormal T-cell clone. ISCL recommends that patients without erythroderma but with blood findings of SS should not be diagnosed as having SS and instead be designated as MF with leukemic involvement. 

Patients with a definitive diagnosis of MF/SS are classified based on the TNMB (tumor, node, metastasis, blood) staging system proposed by ISCL/EORTC (The European Organization for Research and Treatment of Cancer) [9]. The treatment for MF/SS should involve a multidisciplinary treatment team with hematology, dermatology, pathology, and radiation oncology. Due to the rarity of malignancy, patients with Sezary syndrome should be seen in a tertiary care referral center. Patients with limited-stage MF have an excellent prognosis and can be treated with several options including Involved site radiation therapy, phototherapy with ultraviolet (UV), topical corticosteroids, topical retinoids, topical imiquimod, and total skin electron beam therapy [10]. Skin-directed therapies can be offered alone or in combination with other therapies. In advanced stages with skin erythroderma, or with SS, treatment often involves systemic therapy used in combination with skin-directed therapies.

There are several systemic therapy options for advanced MF/SS including bexarotene, brentuximab, methotrexate, mogamulizumab, romidepsin, alemtuzumab, pembrolizumab, pralatrexate, vorinostat, gemcitabine, and liposomal doxorubicin [10,11]. In a trial of physician choice therapy (bexarotene or methotrexate) vs Brentuximab vedotin in CD-30 positive CTCLs, Brentuximab vedotin, antibody-drug conjugate against CD-30, had a superior response rate when compared to physician choice therapy (56.3% vs 12.5%) [12]. In patients with previously treated MF/SS, Mogamulizumab, a recombinant humanized monoclonal antibody against chemokine receptor 4 (CCR-4) had significantly higher progression-free survival over Vorinostat, a histone deacetylase inhibitor - 7.7 months vs 3.1 months [13]. Pembrolizumab, a monoclonal antibody against PD-1 has shown significant anti-tumor activity in heavily pretreated relapsed/refractory MF/SS with an overall response rate of 38%, with a significant durable response [14]. Clinical trial participation should always be strongly encouraged. 

Bexarotene is a synthetic retinoid that selectively activates the retinoid X receptor (RXR). It was FDA-approved for the treatment of CTCL in 1999. We chose bexarotene as the initial therapy for our patient due to the patient’s advanced age and to limit potential side effects from systemic chemotherapy. The most common toxicities associated with bexarotene are hypertriglyceridemia and central hypothyroidism. Myelosuppression is an uncommon side effect. Our patient experienced both hypertriglyceridemia and hypothyroidism but was successfully managed with dose adjustment of statin and thyroid hormone supplementation. 

## Conclusions

Though differential diagnosis for pruritic erythematous skin rash is wide, comprising both benign and malignant etiology, any persistent skin rash that is unresponsive to conservative management should raise suspicion for cutaneous malignancies including cutaneous lymphoma. Prompt diagnosis and early treatment of cutaneous lymphomas will improve outcomes including overall survival. Primary care physicians should have a high index level of suspicion for atypical symptoms including persistent skin rash and should evaluate for other systemic B symptoms, leading to timely diagnosis. Patients with rare cutaneous lymphomas such as SS should be evaluated in tertiary care centers for a multidisciplinary treatment approach.
